# TROP2 methylation and expression in tamoxifen-resistant breast cancer

**DOI:** 10.1186/s12935-018-0589-9

**Published:** 2018-07-06

**Authors:** Stephanie M. Zimmers, Eva P. Browne, Kristin E. Williams, Rahul M. Jawale, Christopher N. Otis, Sallie S. Schneider, Kathleen F. Arcaro

**Affiliations:** 1 Department of Veterinary & Animal Sciences, University of Massachusetts, Amherst, Life Sciences Laboratories, Room 540D, 240 Thatcher Road, Amherst, MA 01003 USA; 2 Pathology Department, Baystate Medical Center, 759 Chestnut Street, Springfield, MA 01199 USA; 3 Biospecimen Resource and Molecular Analysis Facility, Baystate Medical Center, 3601 Main Street, Springfield, MA 01199 USA

**Keywords:** TROP2, DNA methylation, Tamoxifen-resistant breast cancer (or Tamoxifen resistance breast cancer), *TACSTD2*, Human Methylation 450 BeadChip, 5-aza-2′-deoxycytidine (or decitabine or 5-Aza-dC)

## Abstract

**Background:**

The DNA methyltransferase 1 inhibitor, 5-Aza-2′-deoxycytidine (5-Aza-dC) is a potential treatment for breast cancer. However, not all breast tumors will respond similarly to treatment with 5-Aza-dC, and little is known regarding the response of hormone-resistant breast cancers to 5-Aza-dC.

**Methods:**

We demonstrate that 5-Aza-dC-treatment has a stronger effect on an estrogen receptor-negative, Tamoxifen-selected cell line, TMX2-28, than on the estrogen receptor-positive, MCF7, parental cell line. Using data obtained from the HM450 Methylation Bead Chip, pyrosequencing, and RT-qPCR, we identified a panel of genes that are silenced by promoter methylation in TMX2-28 and re-expressed after treatment with 5-Aza-dC.

**Results:**

One of the genes identified, tumor associated calcium signal transducer 2 (*TACSTD2*), is altered by DNA methylation, and there is evidence that in some cancers decreased expression may result in greater proliferation. Analysis of DNA methylation of *TACSTD2* and protein expression of its product, trophoblast antigen protein 2 (TROP2), was extended to a panel of primary (n = 34) and recurrent (n = 34) breast tumors. Stratifying tumors by both recurrence and ER status showed no significant relationship between TROP2 levels and *TACSTD2* methylation. Knocking down *TACSTD2* expression in MCF7 increased proliferation however; re-expressing *TACSTD2* in TMX2-28 did not inhibit proliferation, indicating that *TACSTD2* re-expression alone was insufficient to explain the decreased proliferation observed after treatment with 5-Aza-dC.

**Conclusions:**

These results illustrate the complexity of the TROP2 signaling network. However, TROP2 may be a valid therapeutic target for some cancers. Further studies are needed to identify biomarkers that indicate how TROP2 signaling affects tumor growth and whether targeting TROP2 would be beneficial to the patient.

**Electronic supplementary material:**

The online version of this article (10.1186/s12935-018-0589-9) contains supplementary material, which is available to authorized users.

## Background

DNA methylation, the addition of methyl groups by DNA methyltransferases (DNMTs) to cytosines within CpG dinucleotides, is a key event in tumorigenesis [1, 2]. Although global hypomethylation is characteristic of cancer cells, hypermethylation of CpG islands (CpG-rich regions of the genome approximately 1 kb in length) is also a hallmark of cancer [1, 3]. Hypermethylation of CpG islands in the promoter region of genes frequently leads to transcriptional suppression [4–6]. Breast cancer subtypes show varying levels and patterns of aberrant methylation [2, 7]. Estrogen receptor-positive (ERpos) breast tumors tend to have higher levels of methylation than estrogen receptor-negative (ERneg) subtypes [7–11]. Of interest, recurrence following anti-hormonal therapies can result in resistant tumors that are either ERpos or ERneg and the methylation levels in the ERneg recurrent tumors are higher than the ERpos recurrent tumors [12]. Several genes may be silenced by DNA methylation in hormone-resistant breast cancer [13], and breast cancer cell line models have demonstrated a role of DNA methylation in the development of the resistance [14–16].

Here we use treatment with the DNMT1 inhibitor, 5-Aza-2′-deoxycytidine or decitabine (5-Aza-dC) of Tamoxifen-sensitive (ERpos, MCF7) and -resistant (ERneg, TMX2-28) cell lines to show that inhibition of methylation affects proliferation only in the cell line model for ERneg, Tamoxifen-resistant breast cancer. The cell-line specific effect of 5-Aza-dC agrees with our previous data demonstrating that the ERneg TMX2-28 line has significantly higher methylation than Tamoxifen-selected lines that retained expression of the estrogen receptor [16]. We identified a panel of genes that were silenced by promoter methylation in the TMX2-28 cell line, but not in the parent MCF7 line. We were particularly interested in a change in the promoter methylation of *TACSTD2*, which could be reversed by treatment with 5-Aza-dC. The decrease in methylation of *TACSTD2* was associated with an increase in TROP2, the protein product for *TACSTD2*.

TROP2 (trophoblast antigen protein 2 or tumor-associated calcium signal transducer 2) is a 35.7 kDa transmembrane glycoprotein encoded by the intronless gene *TACSTD2* [17, 18]. As reviewed by McDougall [19], TROP2 is involved in development, normal intracellular signaling and epithelial cancers, where it provides a signal for proliferation and invasion. TROP2 is expressed in many normal tissues and increased TROP2 expression has been reported for many, but not all epithelial cell cancers [20]. In breast cancer, membrane localization of TROP2 has been correlated with a poor prognosis while intracellular TROP2 has been associated with increased survival [21]. TROP2 is silenced by promoter methylation in some tumors and cancer cell lines (bile duct, lung and prostate) [22–24]. However, TROP2 expression has been associated with both inhibition of proliferation [22, 23] and increased growth and metastasis [20, 25–30]. The role of TROP2 in endocrine-resistant breast cancer has not been evaluated previously. Our finding that TROP2 is silenced by promoter methylation in the Tamoxifen-resistant breast cancer cell line prompted us to manipulate *TACSTD2* expression in cell lines. While knockdown of *TACSTD2* in MCF7 cells did increase proliferation as expected, re-expression of *TACSTD2* in the TMX2-28 cell line did not inhibit proliferation, suggesting that its role alone was insufficient to explain the decreased proliferation observed after treatment with 5-Aza-dC in Tamoxifen-resistant cells.

## Methods

### Cell culture, RNA and DNA isolation

MCF7 and TMX2-28 cells were cultured, with the omission of antibiotics, as previously described [16]. DNA was isolated with the QIAamp DNA Mini Kit (Qiagen Cat. No. 51,304) as per manufacturer’s instructions and protocols described previously [31, 32]. RNA was isolated using TriReagent (Molecular Research Center, Inc. Cat. No. TR118). Purified DNA and RNA were quantified using a NanoDrop 8000 (Thermo Scientific).

### Proliferation assay

Cells were seeded in a 96-well plate at 2500 cells/well (for 5-Aza-dC exposure) or 5000 cells/well (for *TACSTD2* cloned cell lines) and treated with 2.5 μM 5-Aza-dC (Sigma Aldrich, Cat. No. A3656) or 0.1% DMSO (vehicle control) for 120 h. At 60 h cells were refed with 100 μL of growth media with 5-Aza-dC or DMSO. At the time of the assay, 20 μL of Cell Titer 96^®^ Aqueous One Solution (Promega, Madison, WI) was added to each well containing 100 μL of growth media. The plate was then incubated at 37 °C, 5% CO_2_ for 1.5 h. Absorbance at 490 nm was read on a VersaMax Tunable Microplate reader (Molecular Devices, Sunnyvale, CA). Cell proliferation was quantified as a percentage of the control for each cell line.

### Illumina Human Methylation450 (HM450) BeadChip

DNA samples from control to Aza-treated MCF7 and TMX2-28 cells were sent to the University of Southern California for methylation analysis (HM450 BeadChip; Illumina Cat. No. WG-314-1003). Briefly DNA was quantified using an Alu PCR reaction, bisulfite-treated and quantified by additional PCR reactions prior to running on the array. Then the DNA was enzymatically fragmented at 37 °C for 1 h and precipitated in 100% 2-propanol at 4 °C for 30 min followed by centrifugation at 3000×*g* at 4 °C for 20 min. After resuspension in hybridization buffer of dried pellets, samples were incubated at 48 °C for 1 h followed by 95 °C for 20 min after which the samples were loaded onto the HM450 BeadChip and incubated at 48 °C for 16–24 h. After hybridization of DNA to the primers on the BeadChip, wash buffers were used to remove non-specific and unhybridized DNA. A single-base extension of the hybridized primers was then conducted using labeled nucleotides and the BeadChip was stained with Cy-3 and Cy-5 fluorescent dyes. The BeadChip was then read using the Illumina iScan Reader. Illumina Genome Studio Methylation Module (v 1.9.0) was then used to analyze the image data to determine the efficiency of the reaction. The ratio of the fluorescent signals of methylated to unmethylated sites (beta values) was used to calculate the methylation of interrogated CpG loci.

### Pyrosequencing

The EZ DNA Methylation-Lightning kit (Zymo, Cat. No. D5030) was used to bisulfite treat DNA. PCR primers were designed using the Pyromark Assay Design Software version 1.0 (Qiagen). Bisulfite-treated DNA was then amplified using the EPIK Amplification kit (Bioline, Cat. No. BIO-66025). Gene-specific primers targeting the three CpG sites in the promoter region of *TACSTD2* (NM_002353) (GRCh37 HG19 Map position (MAPINFO) Ch1: 59043255, 59043280 and 59043370) analyzed by the BeadChip were designed. Primers for pyrosequencing: FWD GGTTGGGGTTGGGAAAGAA-3′, REV -Biot-5′-ACCCCACCTCCTACTACAAACCTA-3′, SEQ 5′-GGAAAGAAAGAAAAGGGA-3′. The Pyromark vacuum prep tool (Qiagen) was used to isolate single stranded products for pyrosequencing. The Pyromark Q24 system (Qiagen) was used to perform pyrosequencing reactions according to manufacturer’s protocol (Qiagen). Percent methylation at the interrogated CpG sites was determined using the Pyromark Q24 Software.

### Two-step reverse transcriptase PCR (RT-qPCR)

RNA was reverse transcribed using the High-Capacity cDNA Reverse Transcription Kit (Applied Biosytems) supplemented with the RNase Inhibitor, RNasin (Promega). cDNA was quantified using a NanoDrop 8000 (Thermo Scientific) and diluted to 50 ng/μL. Primers for RT-qPCR were designed to span an exon–exon junction, when possible (*TACSTD2* has only one exon) using Primer-BLAST (NIH) (*ACTB* FWD 5′- GGACTTCGAGCAAGAGATGG -3′, REV 5′- AGCACTGGTTGGCGTACAG -3′; *TACSTD2* (in non-transfected cell lines) FWD 5′- AATGTATCCCCTTTCGGTCC -3′, REV 5′- TCCCGGGTTGTCATACAGAT -3′; *TACSTD2* (in transfected cell lines) FWD 5′- GCCTTCAACCACTCAGACCT -3′, REV 5′- GAGACTCGCCCTTGATGTCC -3′; *CGNL1* FWD 5′- GGCTGAGGAGGAAATCGACA -3′, REV 5′- CTCGGCAGCTTCTTCAGTCTTA -3′). RT-qPCR was conducted using the FastStart Universal SYBR Green Master with Rox Reference Dye (Roche) on the Stratagene MxPro (Mx3005P, Agilent). Relative mRNA expression was quantified using the standard curve method normalized to beta-actin.

### Human tissue

Following Institutional Review Board (IRB) approval from Baystate Medical Center (Springfield, MA), HM450 data were collected for 70 of the previously described 86 samples [12]. The 70 tumors included 18 paired tumors (primary and recurrent) from women with an ERpos primary tumor: 12 paired tumors from women with ERpos primary and ERpos recurrent tumors and 6 paired tumors from women with ERpos primary and ERneg recurrent tumors (patient and tumor characteristics are summarized in Table 1).

**Table 1:** Patient and tumor characteristics stratified by ER Status of recurrent tumor

	ERpos primary to ERneg (n = 6)	ERpos primary to ERpos (n = 12)		
Patients				
Age (in years) mean (SD), range				
At primary	53 (8.1), 42–65	58 (15.8), 37–84		
At recurrence	60 (6.1), 53–68	65 (15.6), 40–90		
Menopausal n (%)				
At primary	2 (33%)	6 (50%)		
At recurrence	3 (50%)	8 (66.6%)		
TTR (in months) mean (SD), range	82.8 (77.6) 17–216	84.1 (72.5), 12–252		

	ERpos primary (n = 6)	ERneg recurrent (n = 6)	ERpos primary (n = 12)	ERpos recurrent (n = 12)
Tumors				
PR status n (%)				
+	3 (50)	0	11 (91.6)	9 (75)
−	3 (50)	6 (100)	1 (8.3)	3 (25)
HER2 status n (%)				
+	2 (33.3)	3 (50)	1 (8.3)	2 (16.6)
−	4 (66.6)	3 (50)	11 (91.6)	10 (83.4)
Ki67 IHC n (%)				
Low (≤ 15)	6 (100)	4 (66.6)	9 (75)	8 (66.6)
High (> 15)	0	2 (33.3)	3 (25)	4 (33.4)
Tumor grade n (%)^a^				
0	2 (33.3)	2 (33.3)	1 (9.1)	2 (16.7)
1	0	0	3 (27.3)	2 (16.7)
2	3 (50)	0	4 (36.3)	3 (25)
3	1 (16.6)	4 (66.6)	3 (27.3)	5 (41.6)
Tumor type n (%)				
DCIS	2 (33.3)	2 (33.3)	1 (8.3)	2 (16.7)
IDC	3 (50)	4 (66.6)	8 (66.7)	6 (50)
ILC	0	0	2 (16.7)	3 (25)
IDLC	1 (16.6)	0	1 (8.3)	1 (8.3)

See text for scoring of ER, PR, HER2 and Ki67

*TTR* time to recurrence

^a^Indicates that data are missing for some samples; percentages are calculated on the available data

### Immunohistochemistry (IHC)

Cells were seeded on poly-l-lysine-coated slides (Polysciences, Cat. No. 22247, Warrington, PA) at a density of 2 × 10^5^ cells/mL/slide. Cells were maintained at 37 °C, 5% CO_2_ in a humidified incubator overnight and fixed 24 h later. First, the slides were rinsed with cold phosphate-buffered saline solution (PBS) twice and then fixed in ice cold 100% methanol for 10 min. The slides were then allowed to dry and stored at − 20 °C until staining. Immunohistochemical staining was performed at Baystate Medical Center. The slides were stained for TROP2 (Human TROP-2 affinity purified polyclonal antibody, R&D Systems, Cat. No. AF650) using the HRP-DAB Cell and Tissue Staining kit (R&D Systems, Cat. No. CTS008).

Formalin-fixed paraffin-embedded (FFPE) tissue blocks were prepared from the breast tumor samples. The blocks were sectioned (5 μm thick) and placed on slides. Using the UltraView Universal DAB Detection Kit on the BenchMark Ultra platform, the slides were stained for ER, PR and HER2. The slides were stained for TROP2. Hematoxylin and eosin (H&E) slides were prepared and used for tumor verification. The antibodies used for estrogen receptor alpha (ER), progesterone receptor (PR) and epidermal growth factor receptor 2 (HER2) were previously optimized: ER (Ventana anti-estrogen receptor SP1 rabbit monoclonal primary antibody), PR (Ventana anti-progesterone receptor 1E2 rabbit monoclonal primary antibody) and HER2 (Ventana PATHWAY anti-hER2/neu antibody 4B5 rabbit monoclonal antibody). Ethanol was used to dehydrate the slides followed by xylene post-staining and addition of coverslips. The Ki67 data were obtained from the pathology records.

### Review of pathology

Scoring of slides was conducted by one anatomic pathologist (RJ). Slides were scored for immunoreactivity of 5 antigens; approximate number of positive cells was recorded (%) and intensity of immunoreactivity was reported. For ER and PR, Allred scores were recorded ranging from 0 to 8. Tumors with a score of 3 or greater were considered positive for receptor status and tumors were considered HER2 positive when 30% of the cells contained 3+ membrane staining. Ki67 scores ranged from 0 to 100% as the percentage of positive cells within the area of invasive cells. For TROP2, the scale for percent positive cells was 0: negative, 1: 1–33%, 2: 34–66% and 3: 67–100% and the intensity was reported as a score from 0 (negative) to 3 (strong), this was recorded for both membrane-localized TROP2 and cytoplasmic TROP2. The overall score for membrane or cytoplasmic TROP2 was determined by multiplying the percent positive score by the intensity score (range 0–9). Scoring of TROP2 was completed in one session with a single observer documenting records (SZ). One of the tumor samples was scored twice for TROP2 with similar results.

### DNA purification (human tissues)

DNA was purified from formalin-fixed paraffin embedded (FFPE) blocks as previously described [12] using the BiOstic FFPE tissue DNA isolation kit (Mo Bio, Carlsbad, CA). Briefly, the number of sections needed to purify a minimum of 500 ng of DNA was determined using measurements of the H&E stained slides. Tumor sections were prepared for DNA extraction by aligning the marked tumor H&E slide underneath the unstained slide. A sterile needle was used to remove tissue from the slide.

### Plasmids and DNA transfection

The following vectors were received from the Alberti Lab: pSUPER (empty, control vector), hTrop-2siRNA#19 (*TACSTD2* knockdown construct), pΔEYFP-N1 (empty, control vector) and hTrop2 in pΔEYFP-N1 (*TACSTD2* overexpression construct). MCF7 cells were transfected with 5 μg of either pSUPER (empty, control vector) or hTrop2-siRNA#19 (knockdown construct) plus 200 ng of pΔEYFP-N1 (G418 selection) [20]. TMX2-28 cells were transfected with 2 μg of pΔEYFP-N1 (empty, control vector) or pΔEYFP-N1-hTrop2 with SuperFect (Qiagen No. 301305) per manufacturer’s instructions. Cells were maintained in medium (described above) supplemented with 300 μg/mL of G418 disulfate salt (Sigma Aldrich, Cat No. A1720) prior to seeding for colony isolation. Cells were seeded in a 6-well plate at a density of 1000–2000 cells/well and cultured in selective media until colonies were a sufficient size to harvest (approximately 300+ cells). Colonies were isolated using small cloning cylinders (BellCo Glass, Inc., Vineland, NJ), generating the control-vector transfected cell lines (MCF7-Control and TMX2-28-Control), knockdown (MCF7-*TACSTD2*-Kd) and overexpression cell lines (TMX2-28-*TACSTD2*).

### Data analysis

The methylation data obtained from the HM450 BeadChip was analyzed using Genome Studio Methylation Module (v.1.9.0). Detection p-values of < 0.01 were used to select statistically significant CpG site data. Methylation data were exported from Genome Studio and averages were calculated in Excel. Fold change values for each CpG site were calculated by division of average beta values. Additional file 1: Table S1, Additional file 2: Table S2, Additional file 3: Table S3 provide the filtering parameters used for specific analyses. Statistical analyses were conducted using the StatPlus application (v. 5.8.2.0) as follows: RT-qPCR data were assessed using un-paired t-tests, CpG methylation of tumor samples was assessed using Two-way ANOVAs and IHC data were assessed using paired t-tests.

## Results

### 5-Aza-dC treatment differentially alters the behavior and DNA methylation of the Tamoxifen-resistant, TMX2-28, compared to the parental cell line, MCF7

**Fig. 1:**
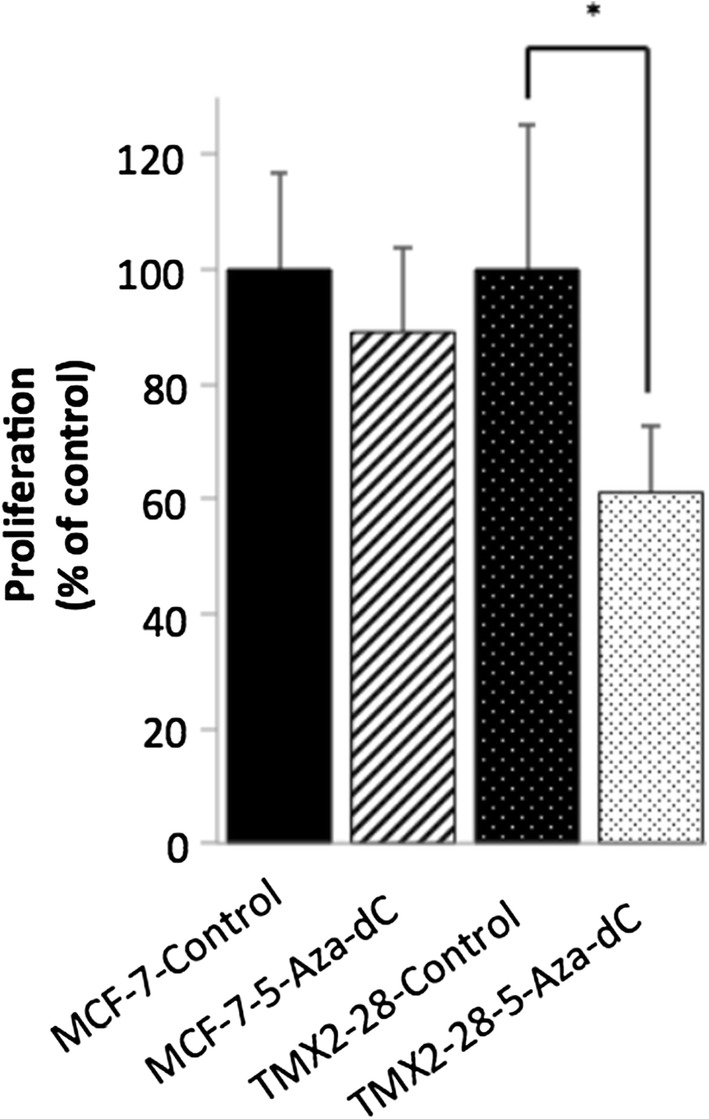
5-Aza-dC treatment decreases proliferation in the Tamoxifen-resistant cell line, TMX2-28. Proliferation, quantified by MTS assay, in control and 120-h 5-Aza-dC-treated cells. Data represented as percentage of control, for the parental line, MCF7, and the ERneg Tamoxifen-resistant cell line, TMX2-28 (n = 8, *p < 0.005)

Using a MTS assay, a colorimetric assay that indicates metabolic activity, treatment with 5-Aza-dC for 120 h was shown to decrease proliferation of TMX2-28 by approximately 39% compared to the DMSO-control-treated TMX2-28. In contrast, treatment of MCF7 with 5-Aza-dC did not alter proliferation (Fig. 1).

**Fig. 2:**
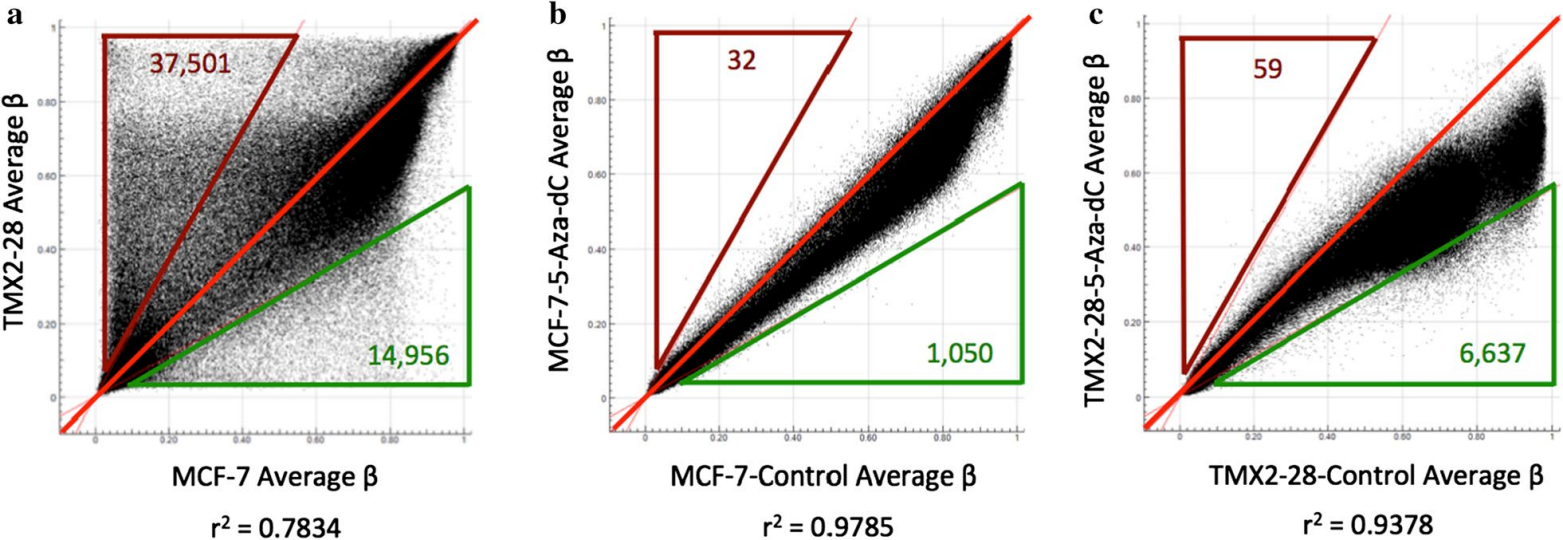
Tamoxifen-resistant cell line is more sensitive than MCF7 to demethylation by 5-Aza-dC treatment. Scatter plots indicating genome-wide methylation differences between the Tamoxifen-resistant cell line, TMX2-28, and the parental line, MCF7 (**a**), between 96-h 5-Aza-dC-treated MCF7 and MCF7-Control (**b**), and between 96-h 5-Aza-dC-treated TMX2-28 and TMX2-28-Control (**c**). Each black dot on the plot represents a CpG site analyzed on the Human Methylation 450 BeadChip. Center red line represents equal average beta values in the two samples and outer red and green triangles indicate the number of CpGs with increased or decreased methylation relative to the cell line on the x-axis, respectively, based on a 1.8-fold change in average beta values

To determine the role of promoter methylation in the differential proliferation of MCF7 and TMX2-28 in response to treatment with 5-Aza-dC, we examined global DNA methylation using the HM450 BeadChip, which interrogates over 450,000 CpGs. We had previously shown that the Tamoxifen-resistant ERneg cell line is significantly hypermethylated throughout the genome as compared to the parental MCF7 [16]. Here we confirm the increased methylation and demonstrate for the first time that the Tamoxifen-resistant cell line is more sensitive to demethylation by treatment with the DNMT1-inhibitor, 5-Aza-dC. Using the filtering parameters provided in Additional file 1: Table S1 there are 37,501 significantly hypermethylated CpGs in TMX2-28 as compared to MCF7, while there are less than half that number of hypomethylated CpGs (14,956). These differentially methylated CpGs are shown in Fig. 2a as dots above and below the outer red lines. Comparison of 96-h 5-Aza-dC-treated cell cultures with control cell cultures shows a greater effect of the DNMT1-inhibitor in TMX2-28 (Fig. 2c) than in MCF7 (Fig. 2b). Using the filtering parameters provided in Additional file 2: Table S2, the 5-Aza-dC-treated TMX2-28 have 6637 CpGs that are significantly less methylated than the TMX2-28 control, and only 59 CpGs that are hypermethylated as compared to the control. In contrast, the 5-Aza-dC-treated MCF7 has only 1050 hypomethylated and 32 hypermethylated CpGs as compared to the MCF7 control. Accordingly, the correlation of the beta values between the treated and control cell cultures is lower for TMX2-28 (r^2^ = 0.94) than for MCF7 (r^2^ = 0.98).

### Identification of genes with increased promoter methylation in the Tamoxifen-resistant cell line that are demethylated by 5-Aza-dC

Our goal is to identify genes in Tamoxifen-resistant breast cancer that may provide novel targets for therapy. We approached this by determining the set of CpGs in the promoter region that are both methylated in the Tamoxifen-selected cell line and demethylated by short-term treatment with 5-Aza-dC, as these CpGs may regulate the expression of genes responsible for the decrease in proliferation observed in the TMX2-28 after treatment with 5-Aza-dC. Using the filtering parameters provided in Additional file 3: Table S3, only 707 of the 37,501 CpG sites with increased methylation in TMX2-28 are significantly decreased after treatment with 5-Aza-dC (Additional file 4: Table S4). To determine the extent to which these CpGs are likely to be involved in regulating gene expression, we determined their distribution in the genome. Of the 707 CpG sites, 251 (35%) are located in the promoter (TSS200 or TSS1500 region), 129 (18%) are in the 5′UTR/1st Exon, 223 (32%) are in the body, 13 (2%) are in the 3′UTR, and 91 (13%) are intergenic (neighborhood locations are shown in Additional file 5: Figure S1 and an overview of the selection process in Additional file 6: Figure S2). The 251 CpG sites in the promoter region that are hypermethylated in TMX2-28 and have decreased methylation after 5-Aza-dC treatment are located in 219 genes, of which 27 genes contain two or more of the CpGs (Table 2 and highlighted in Additional file 4: Table S4). We predicted that these target genes are likely re-expressed after 5-Aza-dC treatment and involved in regulating cell behavior. Using RT-qPCR and mRNA expression arrays we examined the expression of 11 of the 27 genes and determined that 6 of these genes were downregulated in TMX2-28 compared to MCF7. Two of these 6 genes (*TACSTD2* and *CGNL1*) were significantly re-expressed in TMX2-28 after 5-Aza-dC treatment (Table 2).

**Table 2:**
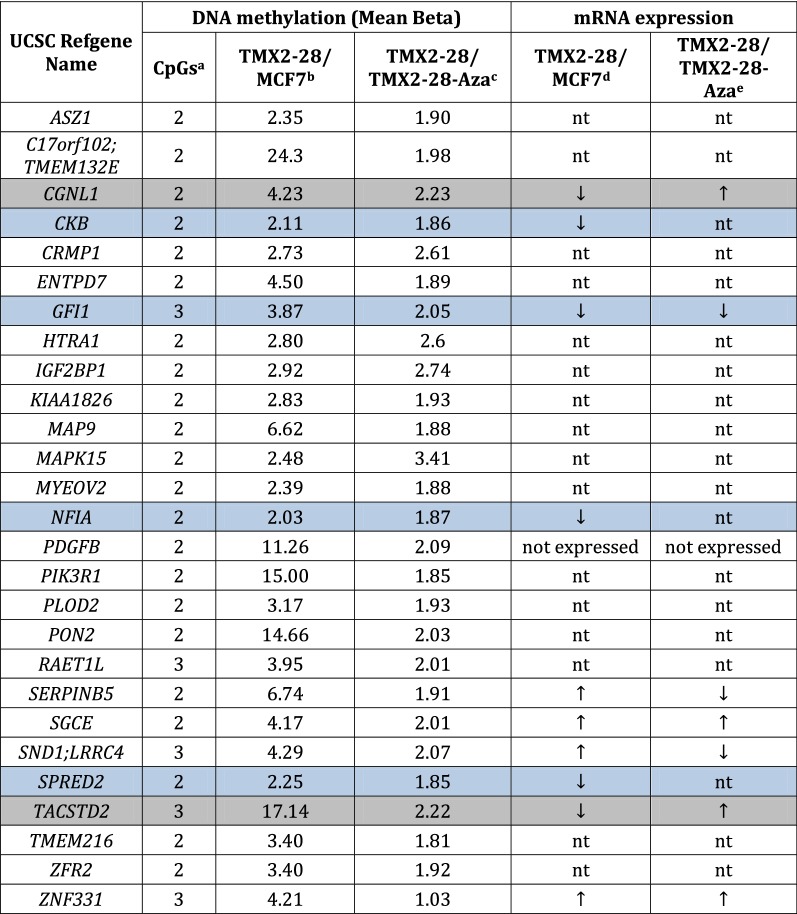
Genes with more than one promoter CpG site hypermethylated in TMX2-28 compared to MCF-7 and with decreased methylation after 5-Aza-dC treatment

Gray shading highlights two genes with decreased expression in TMX2-28 that were re-expressed after treatment with 5-Aza-dC

Blue shading highlights an additional four genes with decreased expression in TMX2-28

*nt* not tested

^a^Number of CpGs in the promoter region that are hypermethylated in TMX2-28

^b^Mean methylation in TMX2-28 compared to MCF7 (fold higher in TMX2-28)

^c^Mean methylation in TMX2-28 compared to TMX2-28 after 72 h treatment with 5-Aza-dC treatment (fold higher in TMX2-28)

^d^Comaprison of mRNA levels in TMX2-28 to MCF7 (direction)

^e^Comparison of mRNA levels in TMX2-28 to TMX2-28 after 72 h treatment with 5-Aza-dC (direction)

**Fig. 3:**
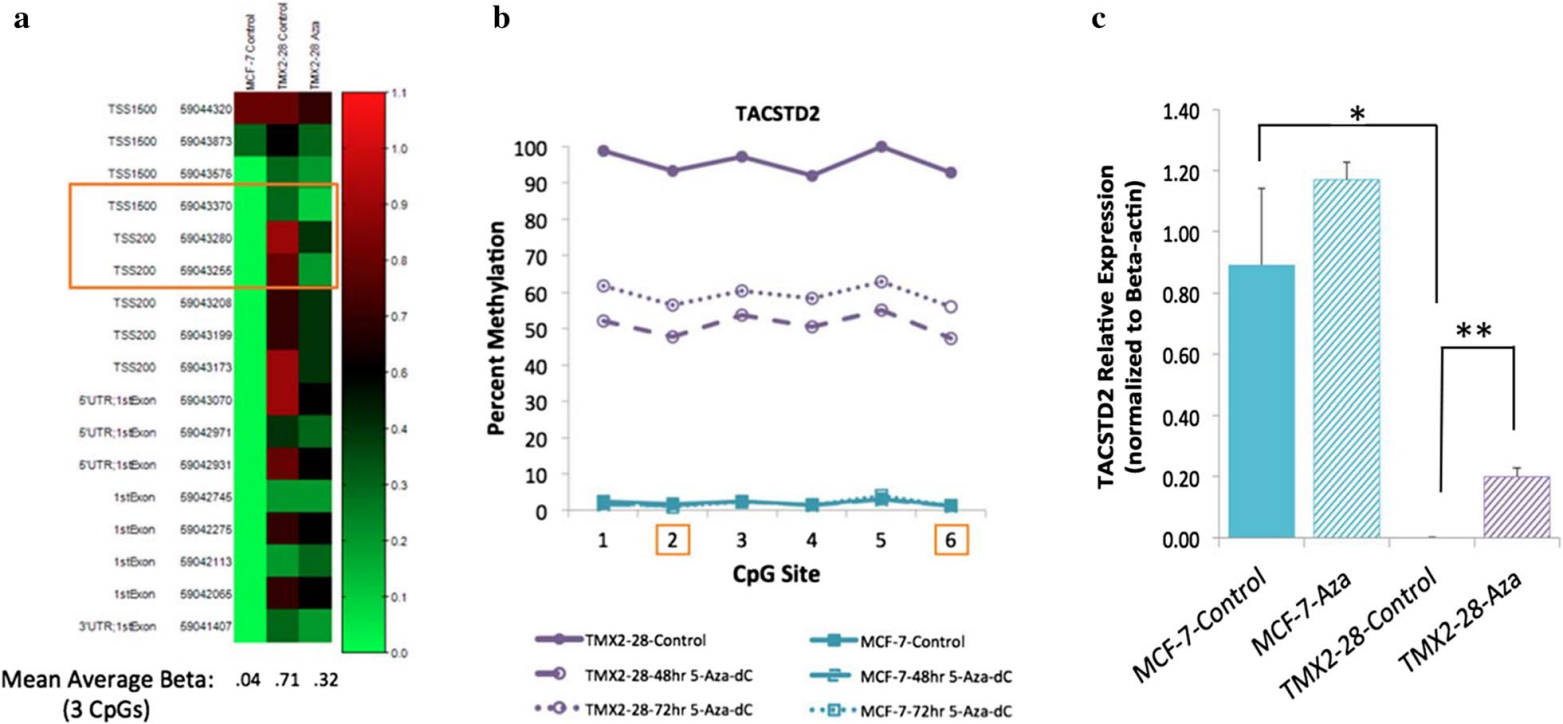
5-Aza-dC decreases *TACSTD2* DNA promoter methylation and is accompanied by increased mRNA expression in TMX2-28. DNA methylation was analyzed with the Illumina Human Methylation 450 BeadChip. Heatmap represents methylation of CpG sites (average beta-values, scale at right) in *TACSTD2* (**a**), for MCF7-Control, TMX2-28-Control and TMX2-28-5-Aza-dC-treated cell lines. Left: Refgene Group location and MAPINFO number for each CpG site. Bottom: Mean average beta values for the 3 CpGs (orange box) identified as hypermethylated in TMX2-28 compared to MCF7 that have decreased methylation in TMX2-28 after 5-Aza-dC treatment. **b** Percent DNA methylation of six CpG sites in the promoter region of *TACSTD2* determined by pyrosequencing (two of which were CpG sites identified as differentially methylated on the HM450 BeadChip, indicated by orange boxes, CpGs 2 and 6 correspond to MAPINFO numbers 59043280 and 59043255, respectively) for control and 5-Aza- -treated cells. Relative *TACSTD2* mRNA expression was quantified by RT-qPCR (**c**) in control or 48-h or 72-h 5-Aza-treated cells, normalized to beta-actin (n = 3, *p < 0.01, **p < 0.001)

We selected one gene, *TACSTD2*, for detailed study. As shown in Fig. 3a, three CpG sites in the promoter of *TACSTD2* were identified as significantly hypermethylated in TMX2-28 as compared to MCF7 and show decreased methylation after treatment with 5-Aza-dC. The mean average beta values for these three CpGs in MCF7-Control, TMX2-28-Control and TMX2-28-5-Aza-dC-treated cell lines are 0.04, 0.71 and 0.32, respectively. Mean methylation of these three CpGs in TMX2-28 decreased by 45% after treatment with 5-Aza-dC but remained significantly higher than MCF7. Next, we confirmed the results from the HM450 BeadChip using pyrosequencing. Interrogation of six CpG sites in the promoter region of *TACSTD2* verified that treatment with 5-Aza-dC, for either 48 or 72 h, decreases *TACSTD2* promoter methylation in TMX2-28 but does not affect methylation in MCF7 (Fig. 3b). We next asked if the decrease in methylation in TMX2-28 results in increased expression. Figure 3c shows that treatment with 5-Aza-dC resulted in a significant increase in mRNA levels of *TACSTD2* in TMX2-28, but not MCF7. The gene chosen for normalization (beta-actin) did not differ between treatment groups (Additional file 7: Figure S3). These results indicate that *TACSTD2* expression is likely regulated by promoter methylation, and support a role of TROP2 in the proliferation differences observed between TMX2-28 and MCF7, as well as a possible role of TROP2 in hormone-resistant breast cancer.

### *TACSTD2* methylation and TROP2 expression in primary and recurrent Tamoxifen-resistant breast cancers

Results from the cell lines suggest that TROP2 may be silenced by promoter methylation in recurrent Tamoxifen-resistant breast cancers, but to date, there are no published studies investigating TROP2 in Tamoxifen-resistant breast cancer. Therefore we used an available DNA methylation data set of 70 primary and recurrent tumors [12] to ask if recurrent breast tumors from women who received anti-estrogen therapy exhibited increased promoter methylation and if methylation was correlated with expression. The data set of 34 primary breast cancers (including 8 primaries that did not recur; 6 ERpos and 2 ERneg), 34 recurrent breast cancers (including 30 first recurrences and 4 second recurrences) and two metastatic breast cancers, included 18 women who had ERpos primary cancers, received anti-estrogen treatment, and had either ERpos (n = 12) or ERneg (n = 6), recurrences. Patient demographics for the 18 women are summarized in Table 1 and for all 70 women are summarized in Additional file 8: Table S5).

**Fig. 4:**
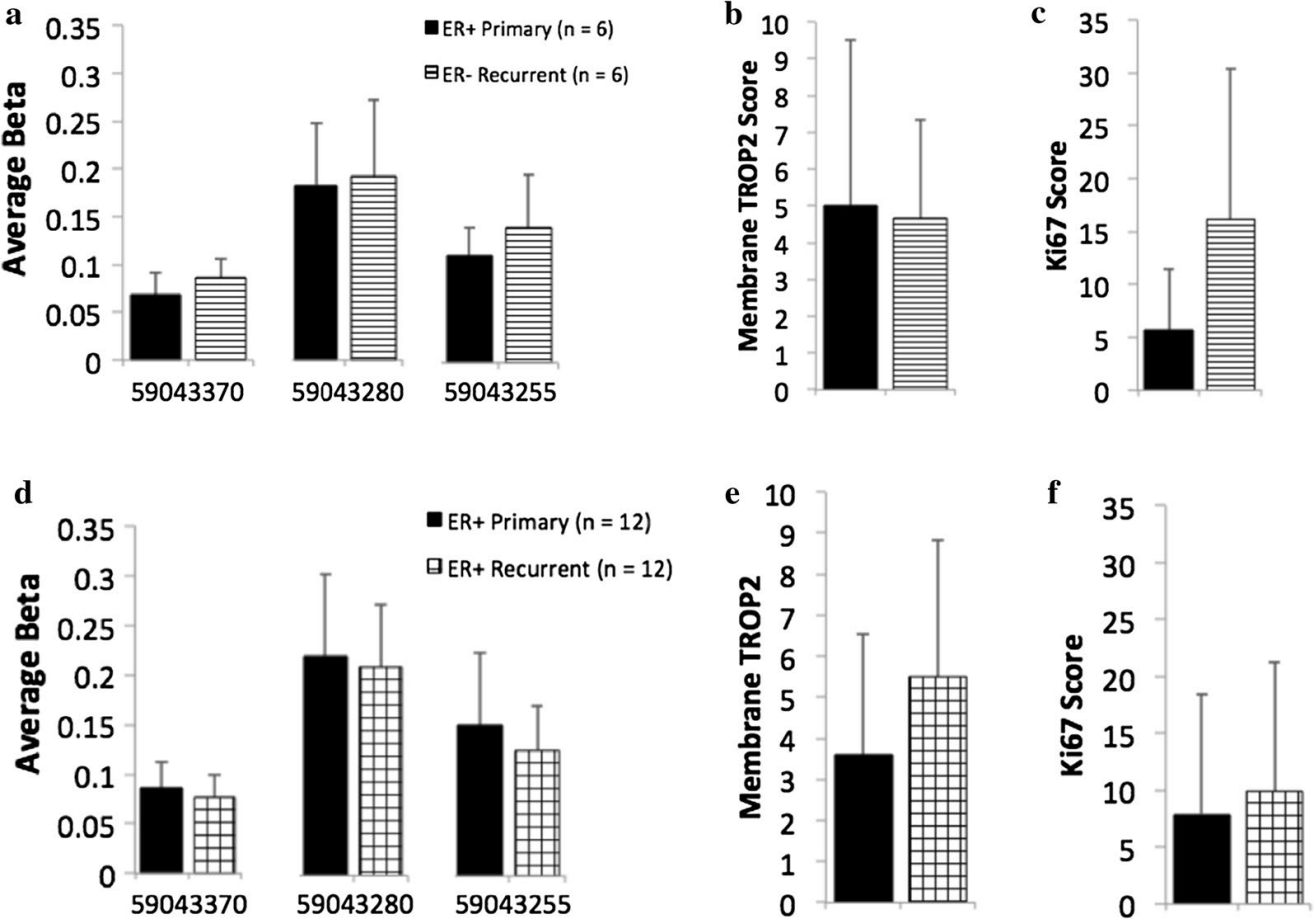
*TACSTD2* methylation and TROP2 staining in primary and recurrent breast tumors. Mean beta values for each of three CpGs in the promoter region of *TACSTD2* (MAPINFO numbers 59043370, 59043280 and 59043255), which were previously identified as hypermethylated in TMX2-28, are shown for 6 sets of ERpos primary to ERneg recurrent breast cancers (**a**), and 12 sets of ERpos primary to ERpos recurrent breast cancers (**d**). There was no significant change in methylation between primary and recurrent tumors when the recurrent tumor was ERneg (**a**: p = 0.25) or ERpos (**d**: p = 0.27). Membrane TROP2 and Ki67 expression, determined by IHC, for same sets of primary and recurrent breast tumors shown in (**b**, **e**) and (**c**, **f**). There was no significant difference in membrane TROP2 expression between primary and recurrent tumor when the recurrent tumor was ERneg (**b**: p = 0.891) and or ERpos (**e**: p = 0.067; n = 10 because staining failed in two recurrent tumors). There was no significant difference in Ki67 expression between primary and recurrent tumor when the recurrent tumor was ERneg (**c**: p = 0.182) or ERpos (**f**: p = 0.463)

Limiting the analyses to those women who received anti-estrogen treatment, comparison of *TACSTD2* promoter methylation (the 3 CpGs that were significantly methylated in TMX2-28) showed no difference between ERpos primary and recurrent tumors (mean beta = 0.142, n = 18 versus 0.138, n = 18 for the primary and recurrent, respectively; see Additional file 9: Table S6). Further stratifying the recurrent tumors by ER status showed no significant increase in methylation of ERneg recurrent tumors (Fig. 4a), or ERpos recurrent tumors (Fig. 4d).

**Fig. 5:**
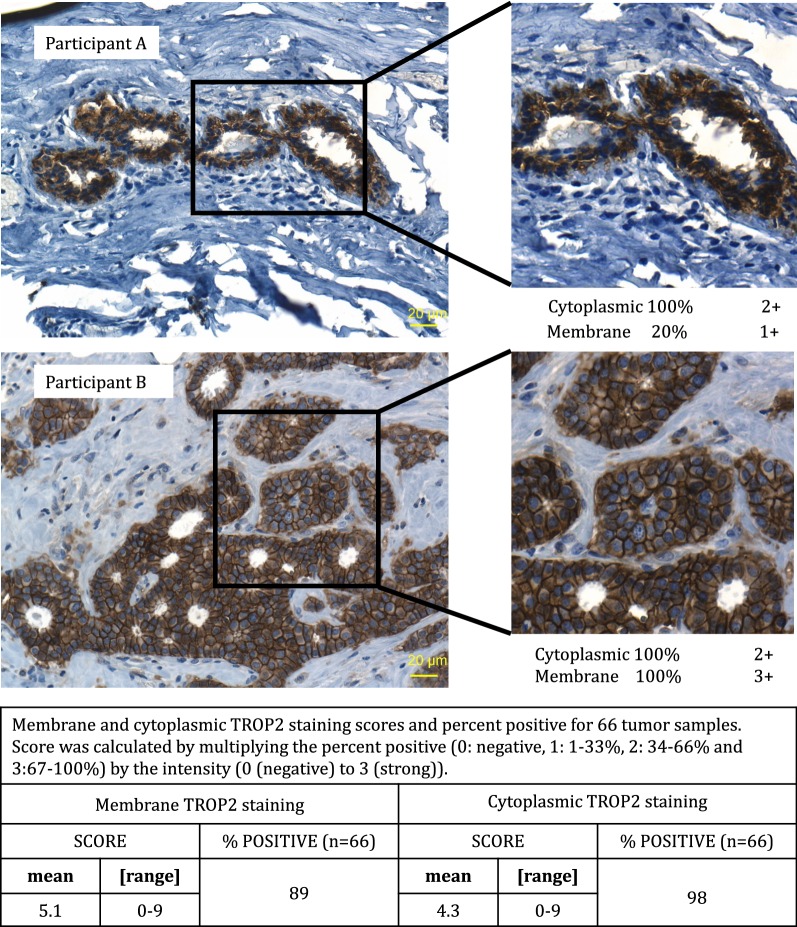
TROP2 scoring in tumor samples. Mean membrane and cytoplasmic TROP2 scores (percentage of positive tumor cells and intensity of staining) determined by IHC for two tumor samples, Participant A and Participant B and summary of staining for all tumor samples. Magnification = 200×

We next asked if the DNA methylation was associated with protein expression. Protein expression and localization (cytoplasmic and/or membrane) of TROP2 were determined in 66 tumor samples by IHC. Examples of cytoplasmic and membrane TROP2 staining are shown in Fig. 5. Analysis of all 66 tumors showed that there was no correlation between the *TACSTD2* methylation (average of the three CpGs) with either cytoplasmic or membrane TROP2 staining (r^2^ = 0.0033 and 0.00063, respectively); nor was there a correlation between methylation and protein staining when the data were stratified by ER status of the primary and recurrent tumors (ERpos primary to ERpos recurrent; n = 12; r^2^ = 0.023 for cytoplasmic; r^2^ = 0.040 for membrane; ERpos primary to ERneg recurrent; n = 6; r^2^ = 0.044 for cytoplasmic; r^2^ = 0.062 for membrane).

Stratifying by ER status of the recurrent tumors, and limiting our analyses to just those women who received anti-estrogen treatment, there was slightly greater TROP2 expression in the ERpos recurrent as compared to the ERneg recurrent (Fig. 4b, e). Considering the small sample size in the subgroups of tumors and the slight increased methylation and decreased expression in ERneg recurrent cancers, it remains feasible that TROP2 plays a role in hormone-resistant breast cancer. Therefore, we further investigated the role of TROP2 in proliferation in the cell lines.

### Proliferation in TMX2-28-*TACSTD2*-expressing and MCF7-*TACSTD2*-knockdown cell lines

**Fig. 6:**
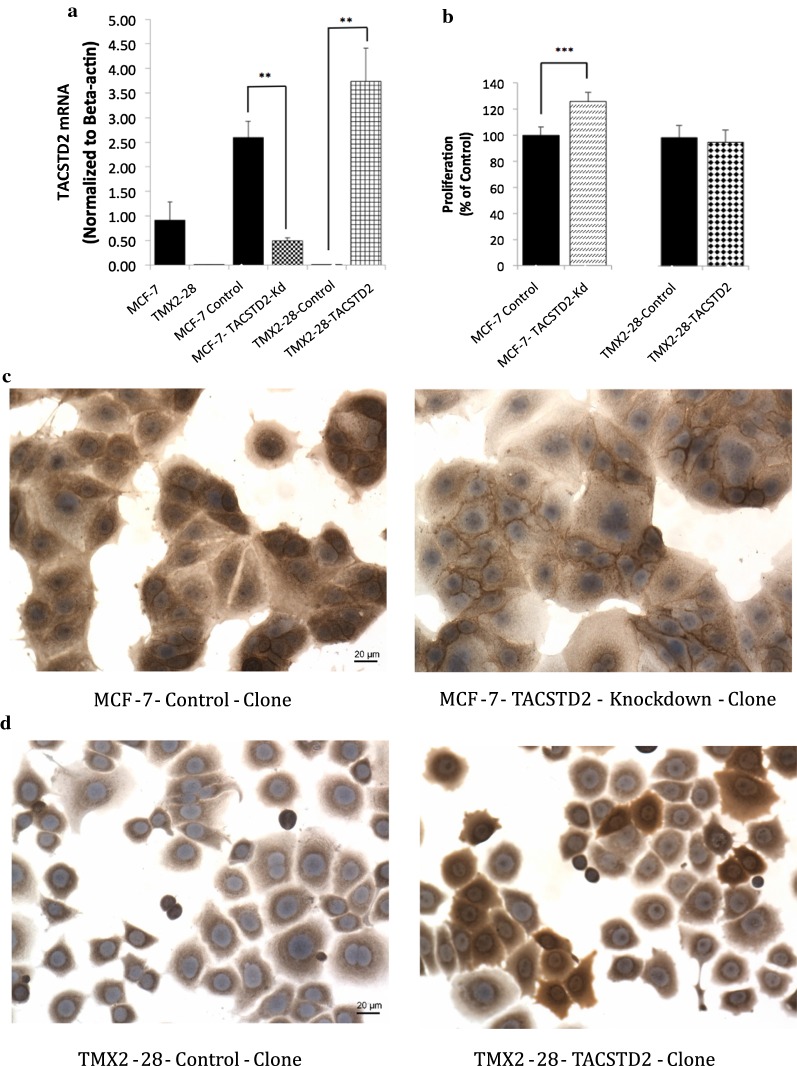
*TACSTD2* expression and proliferation and stable expression of TROP2 in TMX2-28 and knockdown of TROP2 in MCF7. (**a**) *TACSTD2* mRNA expression determined by RT-qPCR run in technical triplicate for MCF7 (non-transfected), MCF7-Control, MCF7-*TACSTD2*-Knockdown, TMX2-28 (non-transfected), TMX2-28-Control and TMX2-28-*TACSTD2* cell lines (**p < 0.001). **b** The MTS assay was used to quantify cell proliferation in MCF7-*TACSTD2*-Kd and TMX2-28-*TACSTD2* cell lines (n = 16), represented as percent of control for each respective cell line (***p < 0.0001). **c** Expression of TROP2 determined by IHC for MCF7-Control-Clone and MCF7-*TACSTD2*-Knockdown-Clone. **d** Expression of TROP2 determined by IHC for TMX2-28-Control-Clone and TMX2-28-*TACSTD2*-Clone. Magnification = 200×

To examine the role of TROP2 in regulating proliferation, stable cell lines were generated with reduced (MCF7-*TACSTD2*-Kd) and increased (TMX2-28-*TACSTD2*) expression of *TACSTD2*. Control cell lines with a vector containing either the scrambled control shRNA (MCF7-Control) or lacking the *TACSTD2* coding sequence (TMX2-28-Control) were also generated. As determined by RT-qPCR, transfection with the shRNA vector against *TACSTD2* resulted in an 80% knockdown of *TACSTD2* mRNA compared to MCF7-Control, and *TACSTD2* mRNA expression increased by more than 100% in TMX2-28-*TACSTD2* (Fig. 6a). Examination of stained cultures by IHC showed decreased TROP2 expression in the MCF7-*TACSTD2*-Kd cell line, and increased expression of TROP2 in the TMX2-28-*TACSTD2* cell line (Fig. 6c and d). If TROP2 plays a major role in the phenotype observed in response to 5-Aza-dC treatment, TMX2-28-*TACSTD2* should exhibit decreased proliferation compared to TMX2-28-Control. In contrast to our expectation, proliferation was not decreased in TMX2-28-*TACSTD2*. Also surprising was the finding that knockdown of *TACSTD2* in MCF7 resulted in a slight increase in proliferation (25%), compared to MCF7-Control (Fig. 6b).

## Discussion

Several studies, including one from our lab, indicate that DNA methylation may be involved in the development of endocrine-resistant breast cancer [13–16]. Therefore, epigenetic therapies that reverse aberrant DNA methylation may prove to be promising therapy for Tamoxifen-resistant disease. However, it is unclear how DNMT inhibitors such as 5-Aza-dC affect methylation and cell behavior, specifically in ERneg, acquired Tamoxifen resistance.

Treatment with 5-Aza-dC inhibited the proliferation of the Tamoxifen-selected, TMX2-28, but not the parental MCF7. TMX2-28 is hypermethylated compared to MCF7 ([12, 16] and Fig. 2) and was more sensitive to inhibition of DNA methylation by treatment with 5-Aza-dC (Fig. 2b and c) suggesting that epigenetic treatment might be particularly beneficial for women with endocrine-resistant breast cancer. The genes identified as methylated in TMX2-28 and demethylated and potentially re-expressed after treatment with 5-Aza-dC may be good biomarkers of sensitivity to epigenetic therapy. We focused on one of these genes, *TACSTD2*, and its product, TROP2, because of its importance as a potential target for treatment.

*TACSTD2* is overexpressed in many cancers and has been associated with disease progression, migration, recurrence, and increased proliferation [27, 33–36]. In published cell culture experiments, knockdown of *TACSTD2* inhibits growth in MCF7 (contrary to our findings) and colon cancer cells [20], fetal rat lung cells (fibroblasts) [28], fetal lung fibroblasts [27], cervical cancer cells [26], and laryngeal carcinoma cells [30]. However, there also is evidence of decreased *TACSTD2* expression in cancer [20, 23, 43, 44]. Lin and colleagues [23] reported that knockdown of *TACSTD2* in lung cancer cells expressing high levels of endogenous *TACSTD2*, increased AKT activation and promoted growth. They further showed that promoter methylation is correlated with decreased *TACSTD2* expression in lung cancer cell lines and tumors [23]. In breast cancer, high levels of membrane TROP2 have been associated with poor prognosis while cytoplasmic staining indicated better survival [21]. Analysis of 2061 ERpos breast cancers using the Kaplan–Meier Plotter indicates that low levels of *TACSTD2* mRNA are associated with decreased survival (p = 0.018; Additional file 10: Figure S4), while analysis of 801 ERneg breast cancers provides the opposite result: low levels of *TACSTD2* are associated with increased survival (p = 0.083; Additional file 10: Figure S4). [37] The extent to which *TACSTD2* mRNA levels in these 2862 ERpos and ERneg breast cancers is associated with membrane versus cytoplasmic TROP2 is unknown. Despite the discrepancies, the evidence for over-expression of *TACSTD2* in cancer is sufficiently compelling to support clinical trials targeting membrane TROP2 [38–45].

## Conclusions

Our results contribute to the growing literature examining the extent to which TROP2 acts as an oncogene or a tumor suppressor. These results also illustrate the complexity of the TROP2 signaling network. Although we found no correlation between promoter methylation and TROP2 expression in patients who received anti-hormone therapy, it would be important to investigate promoter methylation and protein expression in a larger population, as methylation may still play a role in regulating gene expression. Accuracy of IHC scoring may be improved by the use of antibodies specific to cytoplasmic-localized TROP2 and membrane-localized TROP2. Interestingly, we found that primary tumors that did not recur during the time of patient follow-up (approximately 7 years) had higher expression of membrane TROP2 than the ERpos tumors that recurred (data not shown). This also would be important to examine in a larger sample size, as therapies targeted against TROP2 may be unnecessary treatment for some patients. TROP2 may be a valid therapeutic target for some cancers, however, further studies are needed to identify biomarkers that indicate how TROP2 signaling affects tumor growth and whether targeting TROP2 would be beneficial to the patient.

## Additional files

**Additional file 1:Table S1.** Changes in Global Methylation Between Tamoxifen-Resistant Cell Lines and MCF-7.

**Additional file 2: Table S2.** Changes in Global Methylation Between 5-Aza-dC- and Control-Treated Cell Lines.

**Additional file 3: Table S3.** Hypermethylated CpG Sites in TMX2-28 compared to MCF-7 that have decreased methylation after 5-Aza-dC treatment.

**Additional file 4: Table S4.** List of CpGs that are Hypermethylated in TMX2-28 compared to MCF-7 and have Decreased Methylation after 5-Aza-dC Treatment.

**Additional file 5: Figure S1.** Functional genomic location and neighborhood distribution of hypermethylated CpG Sites in TMX2-28 compared to MCF-7 that have decreased methylation after 5-Aza-dC treatment.

**Additional file 6: Figure S2.** Overview of selection process for target genes.

**Additional file 7: Figure S3.** Raw Ct values for β‐actin RT‐qPCR using two different cell lines (MCF7 and TMX2‐28) and different lengths of 5‐Aza‐dC treatment (5‐aza).

**Additional file 8: Table S5.** Patient (n= 70) and tumor characteristics.

**Additional file 9: Table S6.** Beta values for TACSTD2 methylation in clinical samples (ERposprimaries that recur, mean of 3CpGs).

**Additional file 10: Figure S4.** Kaplan Meier plots stratified by ER status.

## Abbreviations

TROP2: trophoblast antigen protein 2
*TACSTD2*: tumor-associated calcium signal transducer 2
5-Aza-dC: 5-aza-2′ deoxycytidine (or decitabine)
ERneg/ERpos: estrogen receptor-negative/positive
DNMT1: DNA methyltransferase 1
FFPE: formalin-fixed paraffin-embedded
IHC: immunohistochemistry
