# Supplementary material for: TROP2 methylation and expression in tamoxifen-resistant breast cancer
Source: Cancer Cell Int. 2018 Jul 6;18:94. doi: 10.1186/s12935-018-0589-9 (PMC6034260; doi:10.1186/s12935-018-0589-9)
Supplement: Supplementary file 1 — Additional file 1:Table S1. Changes in Global Methylation Between Tamoxifen-Resistant Cell Lines and MCF-7. [file 12935_2018_589_MOESM1_ESM.pdf]

Additional file 1: Table S1

Table S1. Changes in Global Methylation Between Tamoxifen-Resistant Cell Lines and MCF-7			
	**Change in Methylation**	**Filter**	**No. of CpG Sites**
**TMX2-28 v. MCF-7**	Hypermethylated	Fold change ≥1.8 in TMX2-28-Control (TMX2-28-Control/MCF-7-Control), β-value ≥0.1 in TMX2-28-Control, Detection p-value ≤ 0.01 for TMX2-28-Control and MCF-7-Control	37,501 (8%)
	Hypomethylated	Fold change ≥1.8 in MCF-7-Control (MCF-7-Control/TMX2-28-Control), β-value ≥0.1 in MCF-7-Control, Detection p-value ≤ 0.01 for TMX2-28-Control and MCF-7-Control	14,956 (3%)
	No Change	Detection p-value ≤ 0.01 for TMX2-28-Control and MCF-7-Control (484,751) minus No. of hypermethylated and hypomethylated CpG sites	432,294 (89%)
