# Supplementary material for: TROP2 methylation and expression in tamoxifen-resistant breast cancer
Source: Cancer Cell Int. 2018 Jul 6;18:94. doi: 10.1186/s12935-018-0589-9 (PMC6034260; doi:10.1186/s12935-018-0589-9)
Supplement: Supplementary file 2 — Additional file 2: Table S2. Changes in Global Methylation Between 5-Aza-dC- and Control-Treated Cell Lines. [file 12935_2018_589_MOESM2_ESM.pdf]

Additional file 2: Table S2

**Table S2. Changes in Global Methylation Between 5-Aza-dC- and Control-Treated Cell Lines**			
	**Change in Methylation**	**Filter**	**No. of CpG Sites**
**TMX2-28-5-Aza-dC v. TMX2-28-Control**	Hypermethylated	Fold change ≥1.8 in TMX2-28-Aza (TMX2-28-5-Aza-dC/TMX2-28-Control), β-value ≥0.1 in TMX2-28-5-Aza-dC, Detection p-value ≤ 0.01 for TMX2-28-5-Aza-dCand TMX2-28-Control	59 (0.01%)
	Hypomethylated	Fold change ≥1.8 in TMX2-28-Control (TMX2-28-Control/TMX2-28-5-Aza-dC), β-value ≥0.1 in TMX2-28-Control, Detection p-value ≤ 0.01 for TMX2-28-5-Aza-dC and TMX2-28-Control	6,637 (1%)
	No Change	Detection p-value ≤ 0.01 for TMX2-28-Control and TMX2-28-5-Aza-dC (484,841) minus No. of hypermethylated and hypomethylated CpG sites	478,145 (99%)
**MCF-7-5-Aza-dC v. MCF-7-Control**	Hypermethylated	Fold change ≥1.8 in MCF-7-5-Aza-dC (MCF-7-5-Aza-dC/MCF-7-Control), β-value ≥0.1 in MCF-7-5-Aza-dC, Detection p-value ≤ 0.01 for MCF-7-5-Aza-dC And MCF-7-Control	32 (0.01%)
	Hypomethylated	Fold change ≥1.8 in MCF-7-Control (MCF-7-Control/ MCF-7-5-Aza-dC), β-value ≥0.1 in MCF-7-Control, Detection p-value ≤ 0.01 for MCF-7-5-Aza-dC and MCF-7-Control	1,050 (0.2%)
	No Change	Detection p-value ≤ 0.01 for MCF-7-Control and MCF-7-5-Aza-dC (485,577) minus No. of hypermethylated and hypomethylated CpG sites	484,495 (99%)
