# Supplementary material for: TROP2 methylation and expression in tamoxifen-resistant breast cancer
Source: Cancer Cell Int. 2018 Jul 6;18:94. doi: 10.1186/s12935-018-0589-9 (PMC6034260; doi:10.1186/s12935-018-0589-9)
Supplement: Supplementary file 3 — Additional file 3: Table S3. Hypermethylated CpG Sites in TMX2-28 compared to MCF-7 that have decreased methylation after 5-Aza-dC treatment. [file 12935_2018_589_MOESM3_ESM.pdf]

Additional file 3: Table S3

**Table S3. Hypermethylated CpG Sites in TMX2-28 compared to MCF-7 that have decreased methylation after 5-Aza-dC treatment**		
**Change in Methylation**	**Filter**	**No. of CpG Sites**
Hypermethylated in TMX2-28 Compared to MCF-7	Fold change ≥1.8 in TMX2-28-Control (TMX2-28-Control/MCF-7-Control), β-value ≥0.1 in TMX2-28-Control, Detection p-value ≤ 0.01 for TMX2-28-Control and MCF-7-Control	37,501
Decreased Methylation in TMX2-28 after 5-Aza-dC	Filter criteria from above and Fold change ≥1.8 in TMX2-28-Control (TMX2-28-Control/TMX2-28-5-Aza-dC), β-value ≥0.1 in TMX2-28-Control, Detection p-value ≤ 0.01 for TMX2-28-5-Aza-dC	707
