# Supplementary material for: TROP2 methylation and expression in tamoxifen-resistant breast cancer
Source: Cancer Cell Int. 2018 Jul 6;18:94. doi: 10.1186/s12935-018-0589-9 (PMC6034260; doi:10.1186/s12935-018-0589-9)
Supplement: Supplementary file 5 — Additional file 5: Figure S1. Functional genomic location and neighborhood distribution of hypermethylated CpG Sites in TMX2-28 compared to MCF-7 that have decreased methylation after 5-Aza-dC treatment. [file 12935_2018_589_MOESM5_ESM.pdf]

Additional file 5: Figure S1

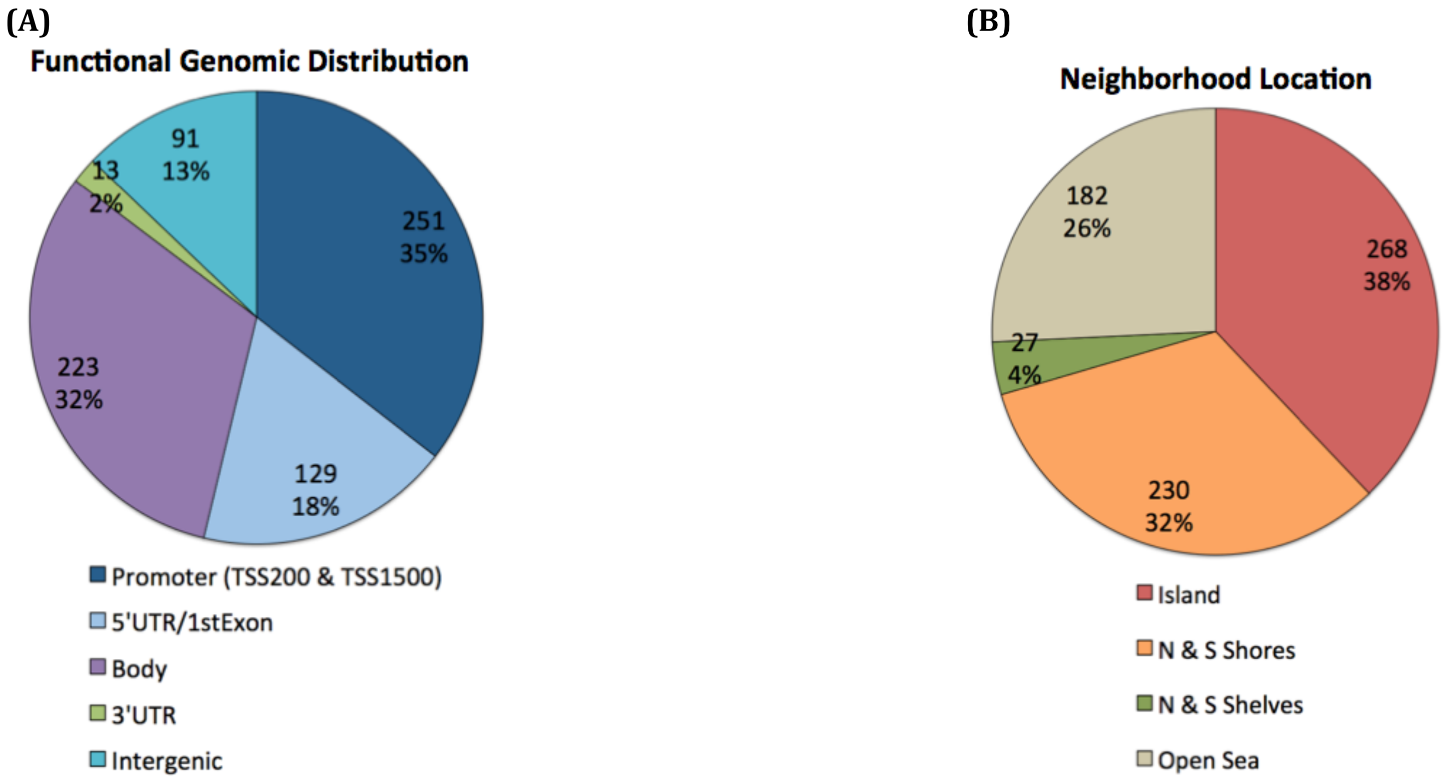

Figure S1: Functional genomic location and neighborhood distribution of hypermethylated CpG Sites in TMX2-28 compared to MCF-7 that have decreased methylation after 5-Aza-dC treatment. Pie charts indicate the functional genomic distribution (A) and neighborhood locations (B) defined by Illumina for the 707 CpG sites identified as having increased methylation in TMX2-28 compared to MCF-7 and also decreased methylation in TMX2-28 after 5-Aza-dC treatment.
