# Supplementary material for: TROP2 methylation and expression in tamoxifen-resistant breast cancer
Source: Cancer Cell Int. 2018 Jul 6;18:94. doi: 10.1186/s12935-018-0589-9 (PMC6034260; doi:10.1186/s12935-018-0589-9)
Supplement: Supplementary file 6 — Additional file 6: Figure S2. Overview of selection process for target genes. [file 12935_2018_589_MOESM6_ESM.pdf]

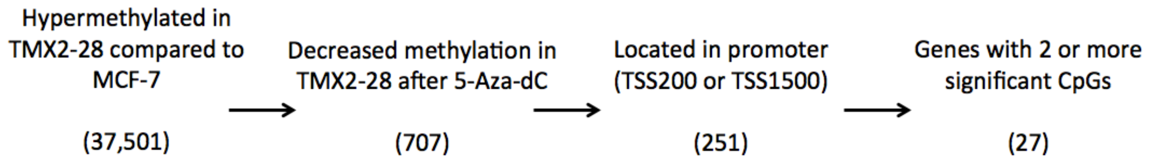

Figure S2. Overview of selection process for target genes. Flow chart describing the selection process and filtering utilized to identify genes with CpG sites located in the promoter that were hypermethylated in TMX2-28 compared to MCF-7 and had decreased methylation when TMX2-28 cells were treated with 5-Aza-dC. Number of CpG sites indicated in parentheses.
