# Supplementary material for: TROP2 methylation and expression in tamoxifen-resistant breast cancer
Source: Cancer Cell Int. 2018 Jul 6;18:94. doi: 10.1186/s12935-018-0589-9 (PMC6034260; doi:10.1186/s12935-018-0589-9)
Supplement: Supplementary file 7 — Additional file 7: Figure S3. Raw Ct values for β‐actin RT‐qPCR using two different cell lines (MCF7 and TMX2‐28) and different lengths of 5‐Aza‐dC treatment (5‐aza). [file 12935_2018_589_MOESM7_ESM.pptx]

## Slide 1
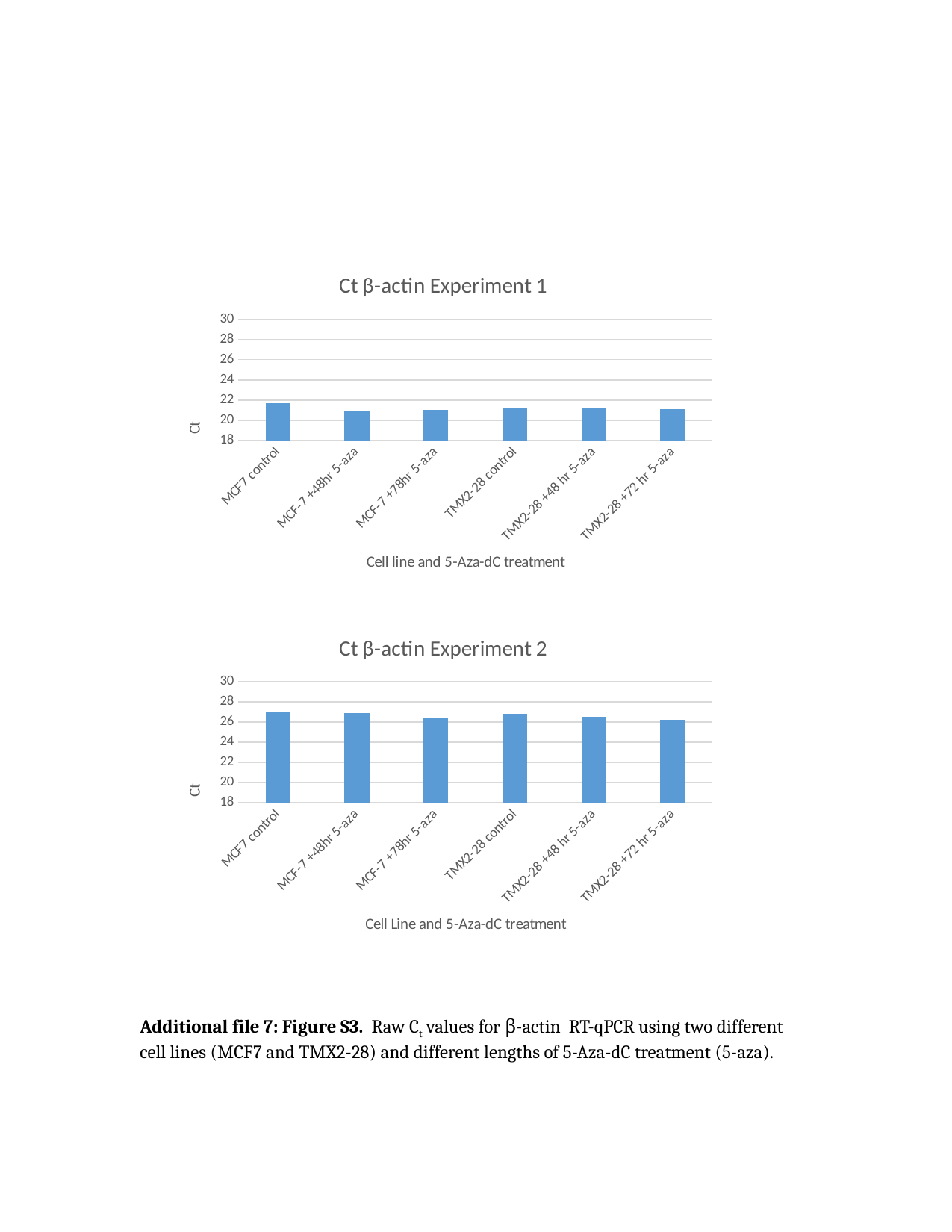

### Chart: Ct β-actin Experiment 1
| Category | C(t) B-actin |
|---|---|
| MCF7 control | 21.665 |
| MCF-7 +48hr 5-aza | 20.925 |
| MCF-7 +78hr 5-aza | 21.015 |
| TMX2-28 control | 21.21 |
| TMX2-28 +48 hr 5-aza | 21.17 |
| TMX2-28 +72 hr 5-aza | 21.105 |
### Chart: Ct β-actin Experiment 2
| Category | C(t) B-actin |
|---|---|
| MCF7 control | 27.045 |
| MCF-7 +48hr 5-aza | 26.92 |
| MCF-7 +78hr 5-aza | 26.48 |
| TMX2-28 control | 26.81 |
| TMX2-28 +48 hr 5-aza | 26.555 |
| TMX2-28 +72 hr 5-aza | 26.215 |Additional file 7: Figure S3. Raw Ct values for β-actin RT-qPCR using two different cell lines (MCF7 and TMX2-28) and different lengths of 5-Aza-dC treatment (5-aza).
