# Supplementary material for: TROP2 methylation and expression in tamoxifen-resistant breast cancer
Source: Cancer Cell Int. 2018 Jul 6;18:94. doi: 10.1186/s12935-018-0589-9 (PMC6034260; doi:10.1186/s12935-018-0589-9)
Supplement: Supplementary file 8 — Additional file 8: Table S5. Patient (n= 70) and tumor characteristics. [file 12935_2018_589_MOESM8_ESM.pdf]

Additional file 8: Table S5

**Table S5. Patient (n= 70) and tumor characteristics**		
**Age** (in years) mean (SD), range		
At Primary	56.6 (12.3), 37 - 84	
At Recurrence	64.1 (12.6), 39 - 90	
**Menopausal** n (%)		
At Primary	13 (38%)	
At Recurrence	21 (58%)	
**TTR**[1] (in months) mean (SD), range	67.8 (59.5) 10-252	
**Tumors**		
	**Primary**[2] **(34)**	**Recurrent**[3] **(36)**
**ER status** n (%)		
+	26 (76.5)	19 (52.8)
-	8 (23.5)	17 (47.2)
**PR status** n (%)		
+	20 (58.8)	16 (44.4)
-	14 (41.2)	20 (55.6)
**HER2 status** n (%)		
+	5 (14.7)	9 (25)
-	29 (85.3)	27 (75)
**Ki67 IHC** n (%)		
low (≤15)	26 (76.5)	20 (55.6)
high (>15)	8 (23.5)	16 (44.4)
**Tumor Grade** n (%)[4]		
0	5 (15.1)	5 (15.1)
1	5 (15.1)	2 (6.1)
2	11 (33.3)	5 (15.1)
3	12 (36.3)	21 (63.6)
**Tumor Type** n (%)		
DCIS	5 (14.7)	5 (13.9)
IDC	23 (67.6)	26 (72.2)
ILC	4 (11.8)	4 (11.1)
IDLC	2 (5.9)	1 (2.8)
**Tumor Size** n (%)[4]		
≥20 mm	13 (48.2)	13 (52)
<20 mm	14 (51.8)	12 (48)
**Anti-hormonal Therapy** n (%)[4]		
No	7 (30.4)	NA
Yes, Tam	4 (17.4)	NA
Yes, AI	7 (30.4)	NA
Yes, Tam & AI	5 (21.8)	NA

[1]TTR = time to recurrence; [2]Primary tumors include the 8 non-recurrent tumors
[3]Recurrent tumors include two metastatic tumors; [4]Indicates that data are missing for some samples; percentages are calculated on the available data
