# Supplementary material for: TROP2 methylation and expression in tamoxifen-resistant breast cancer
Source: Cancer Cell Int. 2018 Jul 6;18:94. doi: 10.1186/s12935-018-0589-9 (PMC6034260; doi:10.1186/s12935-018-0589-9)
Supplement: Supplementary file 9 — Additional file 9: Table S6. Beta values for TACSTD2 methylation in clinical samples (ERposprimaries that recur, mean of 3CpGs). [file 12935_2018_589_MOESM9_ESM.pdf]

Additional file 9: Table S6

**Table Sφ. Beta values for TACSTD2 methylation in clinical samples (ER-pos primaries that recur, mean of 3CpGs)**			
**ER-pos Primary Tumor (n = 18)**		**Recurrent Tumor ((ER-pos, n = 12) or (ER-neg, n = 6))**	
Mean	0.14167	Mean	0.13803
Minimum	0.0859	Minimum	0.07132
Maximum	0.26091	Maximum	0.19866
Variance	0.00242	Variance	0.00151
Standard Deviation	0.04924	Standard Deviation	0.03889
