# Supplementary material for: TROP2 methylation and expression in tamoxifen-resistant breast cancer
Source: Cancer Cell Int. 2018 Jul 6;18:94. doi: 10.1186/s12935-018-0589-9 (PMC6034260; doi:10.1186/s12935-018-0589-9)
Supplement: Supplementary file 10 — Additional file 10: Figure S4. Kaplan Meier plots stratified by ER status. [file 12935_2018_589_MOESM10_ESM.pptx]

## Slide 1
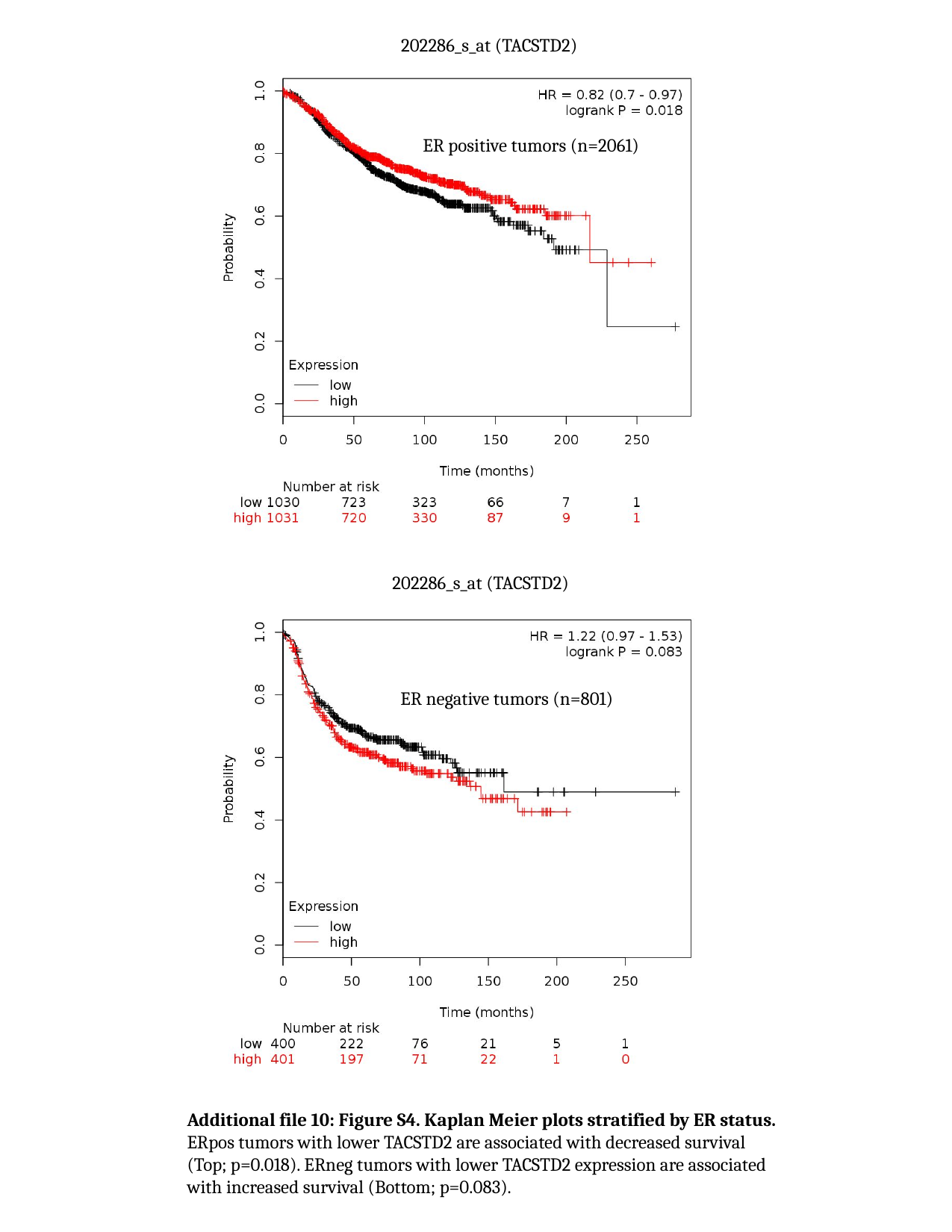

202286_s_at (TACSTD2)
ER positive tumors (n=2061)
	202286_s_at (TACSTD2)
ER negative tumors (n=801)
Additional file 10: Figure S4. Kaplan Meier plots stratified by ER status. ERpos tumors with lower TACSTD2 are associated with decreased survival (Top; p=0.018). ERneg tumors with lower TACSTD2 expression are associated with increased survival (Bottom; p=0.083).
